# Warming Trend: How Climate Shapes *Vibrio* Ecology

**DOI:** 10.1289/ehp.123-A82

**Published:** 2015-04-01

**Authors:** Sharon Levy

**Affiliations:** Sharon Levy, based in Humboldt County, CA, has covered ecology, evolution, and environmental science since 1993. She is at work on *The Marsh Builders*, a book about the history of wetlands and water pollution.

Cholera infects millions of people each year, killing up to 142,000 of its victims.^1^ Localized outbreaks of cholera have been recorded since ancient times; the first documented pandemic began in 1817 in the Ganges River Delta and spread as far as the Middle East and East Africa, resulting in hundreds of thousands of deaths.^2^ A current ongoing pandemic reportedly began in Indonesia in 1961 and has spread to more than 50 countries.^3^

**Figure:**
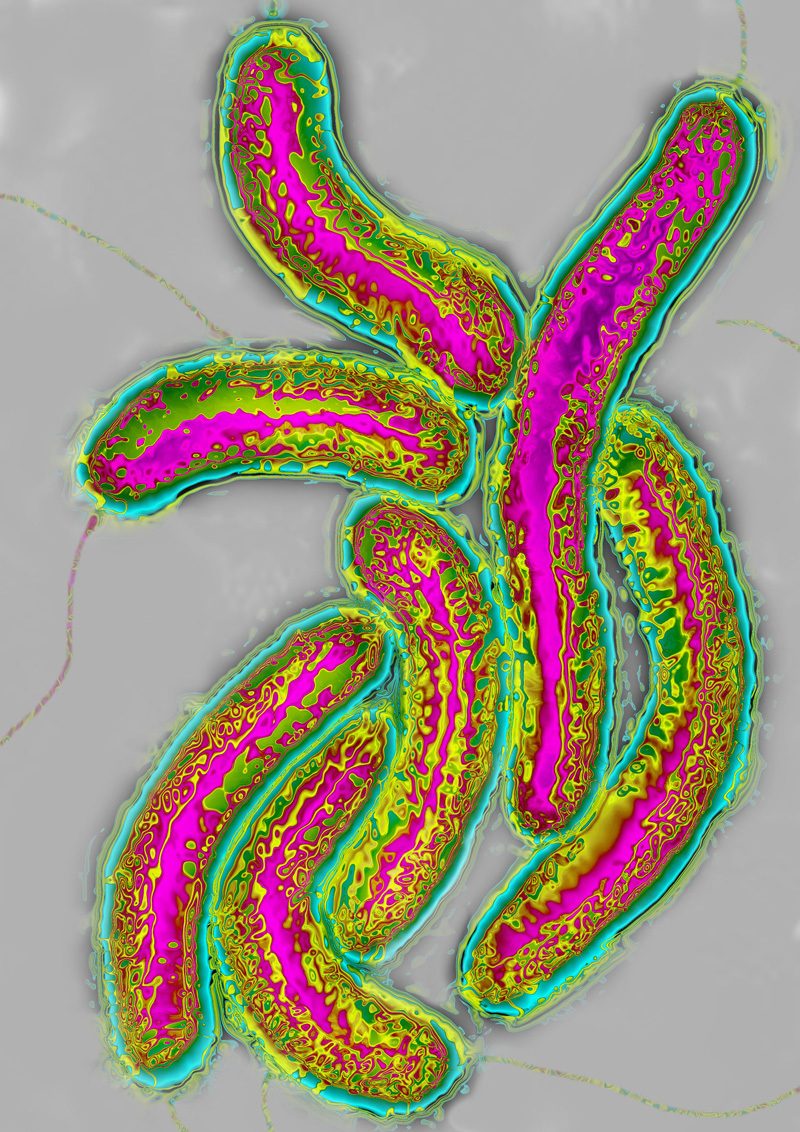
Color-enhanced transmission electron micrograph of Vibrio cholerae, one of multiple pathogenic vibrios whose ecology is closely tied to changes in temperature. © James Cavallini/Science Source

Cholera is caused by the bacterium *Vibrio cholerae*, one of the first pathogenic microbes identified in the nineteenth century. Infection with *V. cholerae* causes acute watery diarrhea that, left untreated, can kill a patient in a single day.^4^ But *V. cholerae* is not the only vibrio, nor is cholera the only vibrionic disease that affects people. *V. parahaemolyticus* and *V. vulnificus* can also cause serious, sometimes fatal, gastrointestinal illnesses and wound infections.

Now a spate of new research is exploring how elements of climate change—from rising air and sea temperatures to intensifying monsoons—affect the ecology of pathogenic vibrios. Recent studies of environmental conditions that lead to cholera outbreaks in the Bay of Bengal and the Indus River Basin offer important insights on the role of climate change in the ongoing clash between human and vibrio.

## The Plankton Connection

Victims can contract cholera by drinking contaminated water, a fact famously demonstrated by Dr. John Snow during an 1854–1855 epidemic in London.^5^ With the rise of sewage treatment and water purification plants during the twentieth century, cholera epidemics vanished from the developed world. But outbreaks continue in places where safe drinking water is unavailable, or when disaster overwhelms sanitation systems.

*V. cholerae* thrive in a commensal relationship with copepods, tiny planktonic crustaceans that drift with the tides and serve as the bacterium’s normal host organism. Rita Colwell of the University of Maryland first demonstrated this relationship in the early 1980s.^6^ She and her colleagues created monoclonal antibodies specific for *V. cholerae* that were tagged with a fluorescent molecule. Under the microscope, the copepods lit up, their bodies outlined in bright dots that signaled the presence of hitchhiking vibrios.

Colwell and many other researchers have delved into the world of *V. cholerae*, discovering its ability to latch onto the chitin in the exoskeletons of copepods, to survive for long periods in a dormant state when copepod populations plummet, and to use chemical signals to communicate and cooperate while infecting a human gut.^7^ “I look at cholera as a vector-borne disease,” says Colwell. “The vector doesn’t fly, it swims; it’s the zooplankton, the copepod.”

**Figure:**
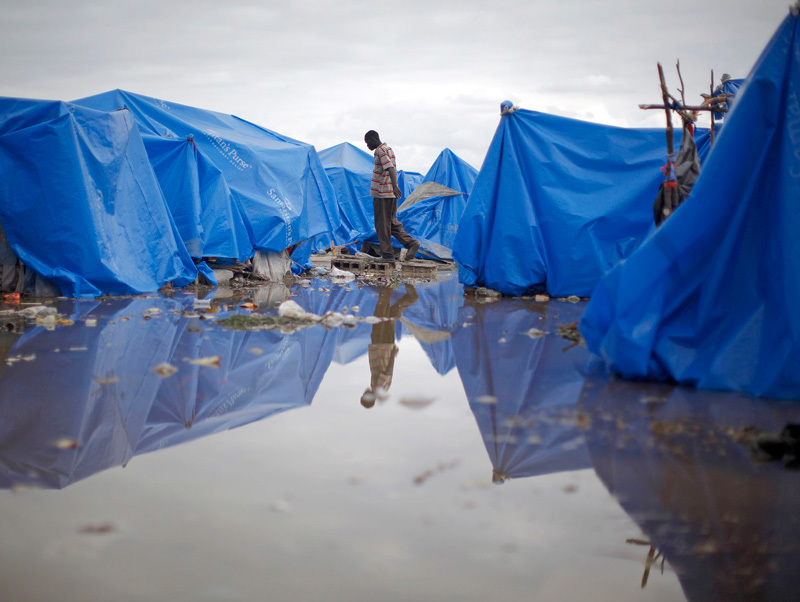
The cholera epidemic that ravaged Haiti after its January 2010 earthquake resulted from a confluence of environmental factors, including unusually heavy rains following an intensely hot summer. © Reuters/Carlos Barria

**Figure:**
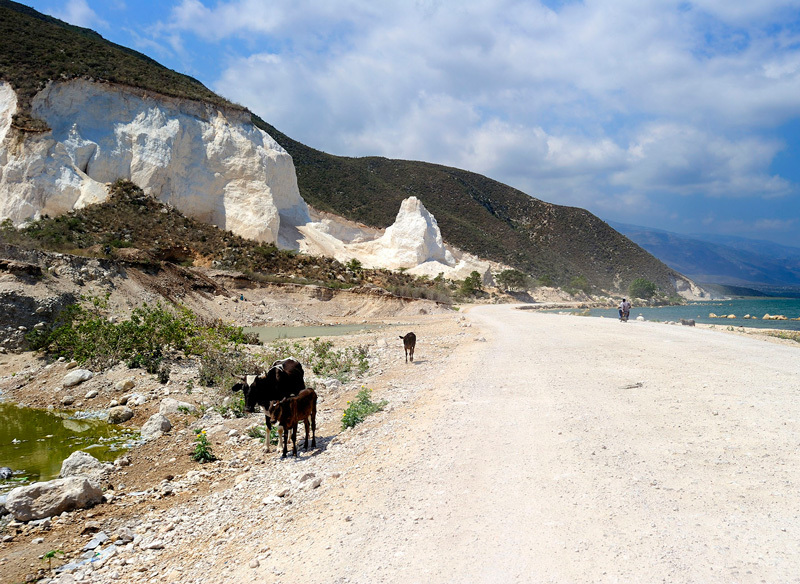
It’s been suggested that the island nation’s limestone foundation may have contributed to the cholera epidemic by increasing the alkalinity of Haiti’s rivers, achieving a pH more favorable for *V. cholerae* growth. © John B. Crane

**Figure:**
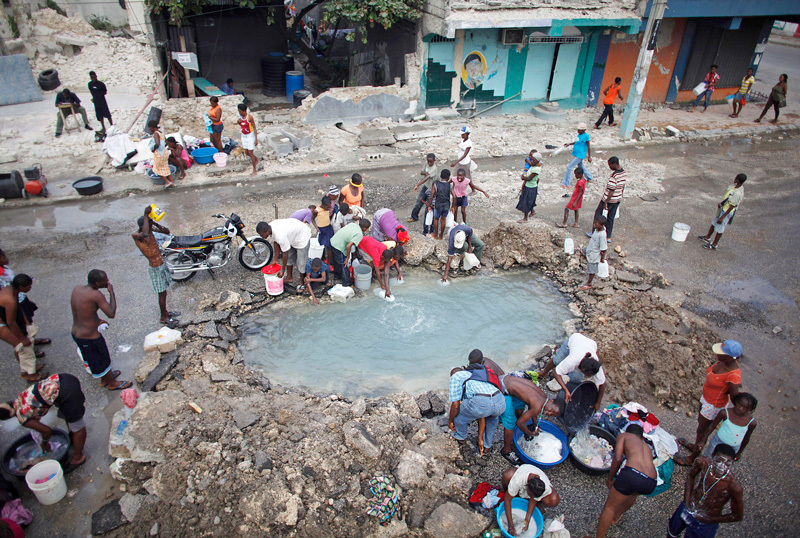
The devastation of drinking water and sanitation systems set the stage for widespread exposure to the pathogenic vibrio. © Reuters/Carlos Barria

Researchers are tracking environmental factors that influence the abundance of copepods. Satellite data on chlorophyll concentrations in the Bay of Bengal revealed that cholera outbreaks in Kolkata, India, and Matlab, Bangladesh—coastal communities where the disease is endemic—were preceded by phytoplankton blooms.^8^ Given *V. cholerae’s* intimate relationship with copepods, this makes sense. A phytoplankton bloom means more food for copepods and other zooplankton; thus, as their copepod hosts become abundant, so do vibrios.

In Bangladesh there are two predictable peaks of infection. The first comes during March and April, a dry time, when the three major rivers that feed the Bengal Delta—the Ganges, the Brahmaputra, and the Meghna—have low flow conditions. This spring outbreak strikes only within 200–300 kilometers of the coast, where bay water from the Bengal Delta pushes upstream, carrying abundant zooplankton and *V. cholerae* into ponds used by local people for domestic water.

The second cholera peak occurs in autumn, generally in September or October, after monsoon rains have boosted river flows. The river waters rush downstream, loaded with nutrients that fuel phytoplankton blooms.^9^ As plankton-rich waters flood the delta, many people are exposed to high concentrations of vibrio bacteria. (A colonized copepod may carry up to 10,000 vibrios.^6^)

In an effort to prevent cholera infections, Colwell and her colleagues taught women in Matlab to filter untreated household water through a folded cloth before using it. An old sari folded into four layers creates a mesh fine enough to capture copepods and particulates, removing 99% of the attached cholera bacteria in the process.^10^ Colwell documented that the local incidence of cholera in Matlab dropped by 48% over a three-year study period,^11^ and followup five years later showed the villagers continued to use this simple technique.^10^

Since the 1960s, Earth’s oceans have absorbed an estimated 90% of the excess heat generated by anthropogenic greenhouse gas emissions.^12^ Sea surface temperatures are rising, with significant impacts on phytoplankton populations.^13^ Phytoplankton abundance has declined in the tropics in recent decades as water temperatures increase—and less phytoplankton should mean fewer copepods and fewer *V. cholerae*, says Shafiqul Islam of Tufts University. But that shift may be countered by a trend toward more extreme drought in dry seasons and heavier rains during the monsoon, as predicted by the Intergovernmental Panel on Climate Change.^14^ “If drought and rainfall become more extreme, cholera will also intensify,” Islam says.

Islam is part of a team of researchers working to understand and predict the environmental drivers of cholera. Using 13 years of satellite data on air temperature and chlorophyll concentrations in the Bay of Bengal, they’ve developed a model that predicts the intensity of both spring and fall cholera outbreaks with 75% accuracy.^3^ They hope to use their growing knowledge of endemic cholera in the Bengal Delta to help predict similar outbreaks that strike throughout the world, which are often far more deadly because people are unprepared.

## Preparing for the Unexpected

In coastal Bangladesh, cholera is familiar, and so is its remedy: drinking a solution of rehydration salts. “Once you have cholera,” says Islam, “you need to hydrate yourself for a few days. ... In a few days, the cholera will get flushed out of your system, and you’re safe. If you don’t rehydrate, you may die.”

**Figure:**
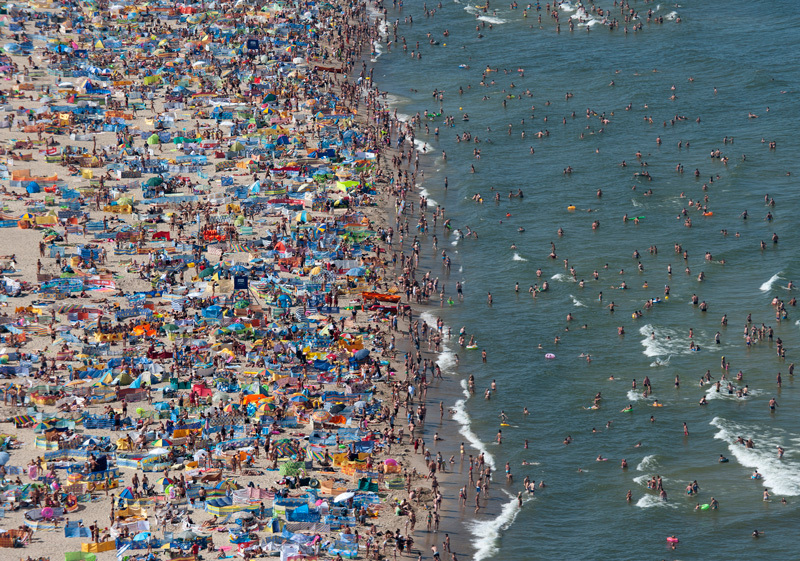
With its low salinity and rising water temperatures, the Baltic Sea is becoming prime habitat for Vibrio species. During the exceptionally hot summer of 2006, at least 66 people developed vibrio infections after visiting Baltic beaches. © Kacper Kowalski/Panos Pictures

But when an epidemic strikes an unprepared region, the death toll can be very high. The ongoing cholera epidemic that first struck Haiti in the aftermath of a devastating 2010 earthquake has had a mortality rate of more than 6%^15^ as compared with 0.01% during a predictable spring or fall outbreak in Bangladesh, where cholera deaths have dropped dramatically in recent decades despite rising infection rates.^16^ Recent epidemics in Zimbabwe, Angola, Nigeria, and Sudan all caused death rates above 3%.^15^^,^^17^

Predictable endemic cholera is a phenomenon of tropical coasts. In contrast, places that suffer massive epidemics at unexpected times are often inland, away from major river deltas. To gather clues to the environmental factors involved in cholera in these epidemic regions, Colwell and Antarpreet Jutla of West Virginia University studied historical data on air temperature, rainfall, and cholera outbreaks in India’s Indus River Basin from 1875 to 1900.^17^ They found that epidemics were strongly predicted by a particular weather pattern: an unusually hot summer followed by heavy rainfall in the fall. High temperatures favor the growth of *V. cholerae* in local waters that spread across the landscape during flooding.

“The historical pattern from the Indus River Basin holds up in other places,” notes Jutla. Increased numbers of cholera outbreaks following heavy rainfall have been recorded in Haiti and Africa.^15^^,^^17^ A comparison of two major flood events in Pakistan points up the powerful influence of weather. A disastrous earthquake in 2005 did not bring a significant outbreak of cholera, because it happened high in the mountains, where cold temperatures discouraged the growth of *V. cholerae*. But in the hot August of 2010, when a flood struck Pakistan’s lowlands, 600,000 people sought treatment for diarrheal diseases, including cholera.^17^

In Haiti, the strain of *V. cholerae* that caused the post-earthquake epidemic is reported to have been introduced by Nepalese United Nations peacekeepers who came to help with disaster relief.^18^ Colwell points out, however, that the epidemic was also preceded by a perfect storm of environmental conditions. *V. cholerae* thrives in alkaline waters, and Colwell hypothesizes that the earthquake may have battered the island’s limestone foundation, releasing ground-up rock into the rivers and increasing the pH. An intensely hot summer was then followed by the greatest rainfall in 50 years.^17^ Drinking water and sanitation systems were devastated by the earthquake. In Colwell’s view, cholera emerged in Haiti because of a complex array of environmental conditions—it was not a simple case of the disease being carried from one part of the world to another inside a human gut.

**Figure:**
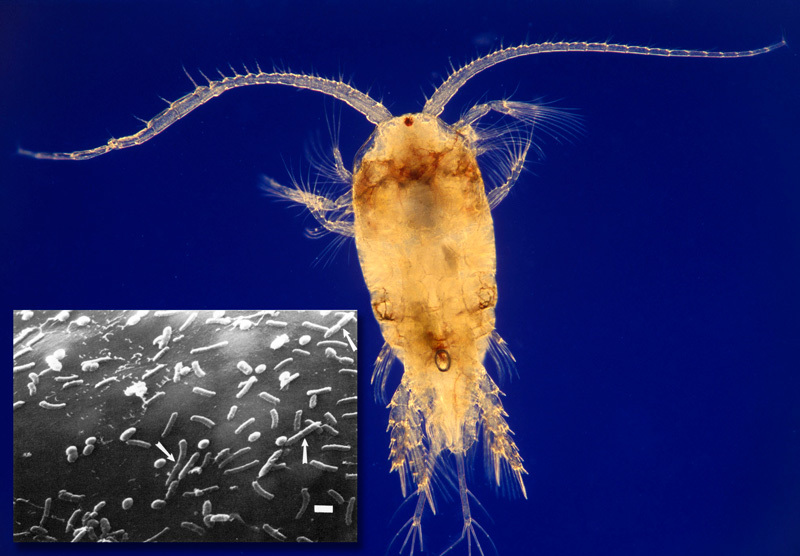
Planktonic organisms called copepods are the natural hosts for vibrios. The inset shows *V. cholerae* dividing on the surface of a copepod egg sac (arrows). A single copepod may carry 10,000 vibrios. *Copepod: © Albert Lleal/Minden Pictures/Corbis; *V. cholerae*: Huq et al. (1983)^6^*

## Warming Conditions

Vibrios live in oceans and estuaries around the world, often without affecting human health; many strains are nonpathogenic or never come into contact with people. “For some kinds of infectious disease agents, crossing a temperature threshold is important,” explains John Balbus, a senior advisor for public health at the National Institute of Environmental Health Sciences. “When temperatures rise, we see outbreaks in places where they’ve not occurred before.” In recent years, outbreaks of vibrio-caused disease have begun to strike in unexpected places—regions that have long been too cold to support booming populations of pathogenic vibrios.

**Figure:**
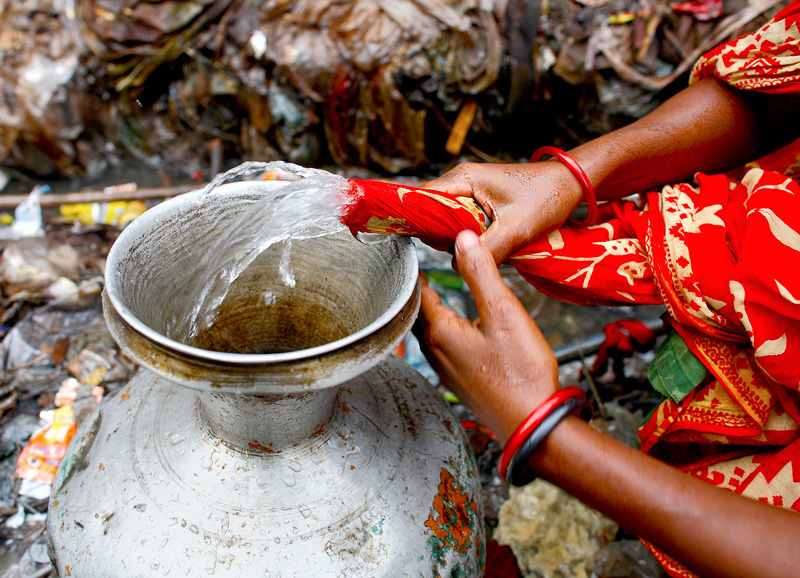
Filtering water through several layers of sari cloth is an easy way to reduce exposure to *V. cholerae*. The layered fabric makes a fine enough mesh to capture copepods—and the vibrios attached to them. © G.M.B. Akash/Panos Pictures

Cyclical fluctuations in weather, such as El Niño events, can powerfully affect the ecology and distribution of vibrios. In coastal Peru, an epidemic of cholera in 1991 and a 1997 outbreak of gastroenteritis caused by *V. parahaemolyticus* were both correlated with strong El Niño currents, which pushed masses of warm water laden with tropical zooplankton and their vibrio communities across the Pacific from Asia to South America. *V. parahaemolyticus* is a common cause of seafood-associated gastroenteritis in warmer parts of the world.

A similar pattern was observed during a 1999 outbreak of gastroenteritis in Galicia, in northwest Spain.^19^ A recent study of vibrio ecology in the waters off Galicia found that *V. parahaemolyticus* showed up in 80% of the zooplankton samples collected.^20^ Warmer water temperatures appear to increase the ability of vibrios to attach to and digest the chitinous carapaces of zooplankton.^12^

In the summer of 2004 an outbreak of diarrheal disease struck several cruise ships sailing in the waters of Prince William Sound, Alaska. The problem was traced to local raw oysters that were contaminated with *V. parahaemolyticus*. For an extended period that summer, water temperatures at the oyster farm had risen above 15°C (59°F)—the temperature at which risk of *V. parahaemolyticus* infection increases. The outbreak expanded the range of confirmed human infection with the pathogen to a latitude higher than 60°N, more than 1,000 kilometers north of previous recorded outbreaks.^21^

The Baltic Sea is prime habitat for *Vibrio s*pecies that are becoming more dominant in northern waters as the oceans warm. Vibrios thrive in warm, low-salinity water. The Baltic is one of the largest low-salinity seas on the planet, and it’s warming fast. Since 1985, summer warming rates have been nearly triple what was forecast for this period of time.^22^

During the extraordinarily hot European summer of 2006, people living near the Baltic Sea flocked to the beaches to swim. At least 66 of them contracted necrotic wound infections caused by vibrios including *V. cholerae* and *V. vulnificus.*^23^ Infection with *V. vulnificus*, a species that was first identified in the 1970s, is rare but serious. Some patients lose the infected limb; if the infection spreads to the bloodstream, it can turn lethal. During recent heat waves, nations bordering the Baltic Sea, including Sweden, Germany, Poland, and Denmark, reported 4 deaths from wound infections with *V. cholerae* and 10 from wounds infected with *V. vulnificus.*^23^^,^^24^

Beyond the extremes of summer heat waves, climate change is also bringing long-term shifts in coastal ecosystems, lengthening the season of warm spring and summer temperatures in which vibrios can flourish. That concept was illustrated in an inventive study that used an archive of preserved plankton samples collected in the North Sea since 1961 to explore changes in vibrio populations. Using a molecular probe that targets DNA of all bacteria in the *Vibrio* genus, the researchers found a major shift in the community of bacteria associated with plankton collected off the Rhine River estuary. A marked increase in vibrio abundance coincided with significant warming in the southern North Sea in the late 1980s.^25^ The increasing dominance of vibrios may signal a decline in microbial biodiversity—a question that remains to be explored.

## Tracking the Impact

“There is now striking evidence that climate change impacts are driving outbreaks of vibrio infection,” says Craig Baker-Austin of the Centre for Environment, Fisheries and Aquaculture Science in Dorset, United Kingdom. “We see a large increase in reported infections during heat wave events.”

**Figure:**
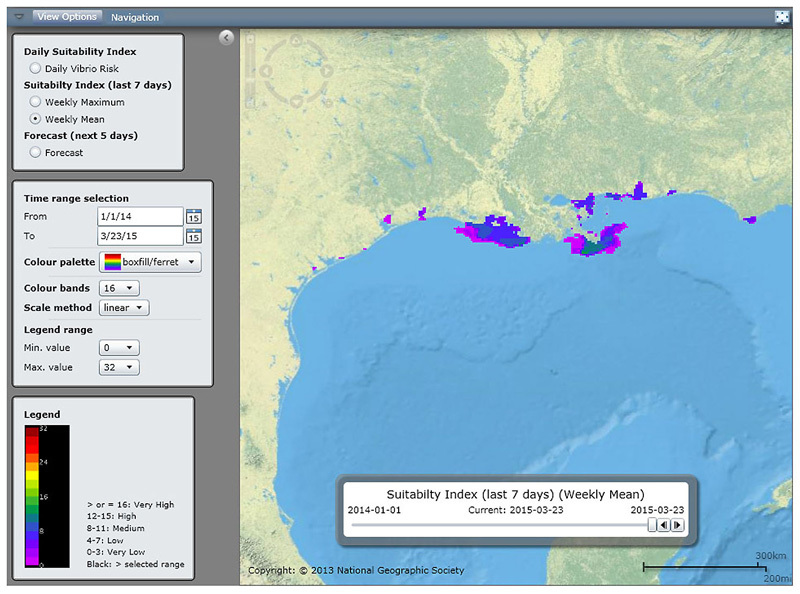
A pilot Vibrio Risk Map on the E3 Geoportal website identifies areas where environmental conditions may be conducive to vibrio growth. The model is updated daily with remotely sensed data on variables such as sea surface temperature and salinity. © European Environment and Epidemiology Network

In the United States, vibrio infections are reported to the Centers for Disease Control and Prevention (CDC). Combining all species, vibrio infections in the United States rose from 387 reported cases in 1997^26^ to 944 reported cases in 2012.^27^ People who are immunocompromised, and those with chronic liver disease are more susceptible to complications from vibrio infection.^28^

Data are harder to track in many European countries, because vibrio infections are not on the list of diseases that must be reported to public health officials. Nevertheless, officials in Northern Europe documented vibrio outbreaks during heat waves in 1994, 1997, 2003, 2006, 2010, and 2014.^23^ Baker-Austin notes that one of the most obvious manifestations of a warming climate is an increase in the frequency and severity of heat waves. In a recent study, he and his colleagues examined long-term data on sea surface temperature in the Baltic and found a clear correlation between episodes of unusually high water temperature and outbreaks of pathogenic vibrio infections.^24^

Baker-Austin believes that even in the United States, where doctors must report vibrio infections to the CDC, the recorded cases represent a fraction of the total number of cases. Many cases of vibrionic gastroenteritis are not severe enough to send people to the doctor—or if they go to the doctor, there may or may not be a stool culture to confirm *Vibrio* infection. “For every laboratory-confirmed infection,” Baker-Austin says, “there are many more circulating in the community.”

Jan Semenza and his colleagues at the European Centre for Disease Prevention and Control (ECDC) have used data on sea surface temperature and salinity, obtained from remote-sensing satellites, to create a real-time model that shows coastal areas that are environmentally suitable for vibrio growth. The Vibrio Risk Map, available through the ECDC’s E3 Geoportal website,^29^ shows coastal areas where sea surface temperatures and salinity levels indicate favorable conditions for *Vibrio* growth. “The purpose of this tool is to alert the public that there is a potential vibrio bloom, a potential health threat,” says Semenza. He hopes the Vibrio Risk Map will be used by health departments around the world as an early warning of vibrio risk; beach closures or warnings directed at the most susceptible people could prevent infections and save lives.

Because vibrio bacteria are a normal part of aquatic ecosystems, they can never be eradicated; we can only manage our exposure. Colwell is reminded of that during her daily jog along the Potomac River. She passes a plaque memorializing the workers who died of cholera while building the Chesapeake & Ohio Canal in the 1830s. The workers, impoverished Irish immigrants, lived in crowded shacks without safe drinking water or sanitation. In the hot summer of 1832, a classic combination of environmental conditions produced an epidemic that killed uncounted workers and caused the survivors to drop their tools and flee.^30^

The United States has kept cholera at bay for more than a century by treating its sewage and drinking water. As climate shifts, the communities most vulnerable to cholera will need clean water and sanitation more than ever. The World Health Organization estimates that 748 million people worldwide currently have no access to safe drinking water, and approximately 1.8 billion use water sources that are contaminated with fecal matter.^31^ Beyond the complexities of vibrio ecology lies the daunting challenge of providing safe water and sanitation to growing human populations in a warming world.
